# Exploring the genetic and adaptive diversity of a pan-Mediterranean crop wild relative: narrow-leafed lupin

**DOI:** 10.1007/s00122-017-3045-7

**Published:** 2018-01-20

**Authors:** Mahsa Mousavi-Derazmahalleh, Philipp E. Bayer, Bruno Nevado, Bhavna Hurgobin, Dmitry Filatov, Andrzej Kilian, Lars G. Kamphuis, Karam B. Singh, Jens D. Berger, James K. Hane, David Edwards, William Erskine, Matthew N. Nelson

**Affiliations:** 1 UWA School of Agriculture and Environment, The University of Western Australia, 35 Stirling Highway, Crawley, WA 6009 Australia; 2 School of Biological Sciences, The University of Western Australia, 35 Stirling Highway, Crawley, WA 6009 Australia; 3 Department of Plant Sciences, University of Oxford, Oxford, OX1 3RB UK; 4 School of Agriculture and Food Sciences, The University of Queensland, Brisbane, QLD 4072 Australia; 5 DArT P/L, GPO Box 3200, Canberra, ACT 2601 Australia; 6 CSIRO Agriculture and Food, Wembley, WA 6913 Australia; 7 The UWA Institute of Agriculture, The University of Western Australia, 35 Stirling Highway, Perth, WA 6009 Australia; 8 CCDM Bioinformatics, Centre for Crop Disease Management, Curtin University, Bentley, WA 6102 Australia; 9 Centre for Plant Genetics and Breeding, The University of Western Australia, 35 Stirling Highway, Crawley, WA 6009 Australia; 10 Natural Capital and Plant Health, Royal Botanic Gardens Kew, Wakehurst Place, Ardingly, West Sussex RH17 6TN UK

## Abstract

**Key message:**

This first pan-Mediterranean analysis of genetic diversity in wild narrow-leafed lupin revealed strong East–West genetic differentiation of populations, an historic eastward migration, and signatures of genetic adaptation to climatic variables.

**Abstract:**

Most grain crops suffer from a narrow genetic base, which limits their potential for adapting to new challenges such as increased stresses associated with climate change. Plant breeders are returning to the wild ancestors of crops and their close relatives to broaden the genetic base of their crops. Understanding the genetic adaptation of these wild relatives will help plant breeders most effectively use available wild diversity. Here, we took narrow-leafed lupin (*Lupinus angustifolius* L.) as a model to understand adaptation in a wild crop ancestor. A set of 142 wild accessions of narrow-leafed lupin from across the Mediterranean basin were subjected to genotyping-by-sequencing using Diversity Arrays Technology. Phylogenetic, linkage disequilibrium and demographic analyses were employed to explore the history of narrow-leafed lupin within the Mediterranean region. We found strong genetic differentiation between accessions from the western and eastern Mediterranean, evidence of an historic West to East migration, and that eastern Mediterranean narrow-leafed lupin experienced a severe and recent genetic bottleneck. We showed that these two populations differ for flowering time as a result of local adaptation, with the West flowering late while the East flowers early. A genome-wide association study identified single nucleotide polymorphism markers associated with climatic adaptation. Resolving the origin of wild narrow-leafed lupin and how its migration has induced adaptation to specific regions of the Mediterranean serves as a useful resource not only for developing narrow-leafed lupin cultivars with greater resilience to a changing climate, but also as a model which can be applied to other legumes.

**Electronic supplementary material:**

The online version of this article (10.1007/s00122-017-3045-7) contains supplementary material, which is available to authorized users.

## Introduction

Agriculture is facing the ‘perfect storm’ of factors that threaten global food security including continued population growth, a rapidly changing climate, reduced water available to agriculture, soil erosion and increasing costs of fertilisers (Abberton et al. 2015; Gomiero 2016). A major concern is the lack of diversity in modern cropping systems to adapt to these challenges. For example, the human population derives more than 50% of its calorific intake from just three crop species (Awika 2011). This is largely reflected in the concentration of research and breeding investments into major crops, which serves to widen the gap between well-resourced major crop species and minor crops (also known as ‘neglected under-utilised species’). A wider range of crop species is required to efficiently exploit all available agricultural environmental niches in a sustainable manner (Abberton et al. 2015). Legumes have a special role in this given their ability to raise soil fertility from biological nitrogen fixation, system sustainability and improve human health (Foyer et al. 2016). Depleted diversity is also apparent within most modern crops relative to their wild progenitors due to historic population bottlenecks associated with domestication, breeding and geographic translocation around the world (Gepts 2010). Therefore, there is a strong need to understand and harness adaptive diversity from the wild ancestors and relatives of crop species (Berger et al. 2013; Maxted et al. 2012).

Evolutionary adaptation is one way that wild species may overcome the changes in their environment. For example, climate change has proven to act as a strong selective agent, driving new directions for natural selection in evolutionary adaptation (Hoffmann and Sgro 2011). Rapid adaptive evolutionary changes in response to climatic fluctuations have been reported in both plants and animals, such as those altering flowering phenology of *Brassica rapa* (Franks et al. 2007) and changes in migratory behaviour of birds (Pulido and Berthold 2010). However, the success of evolutionary adaptation is contingent on the availability of genetic diversity within species and genetic control (heritability) of adaptive traits. A recent study in New World lupin species showed there is more frequent genome-wide adaptation in rapidly diversifying species, as opposed to slowly diversifying species and plant species more generally (Nevado et al. 2016). The genetic basis of local adaptation can be explored using a genome-wide scan approach, as demonstrated for soil salinity adaptation of *Medicago truncatula* populations (Friesen et al. 2010). Migration by natural means or by human intervention (in the case of agricultural species) is another driver of adaptive change, particularly for sessile species such as plants. For example, a genetic differentiation analysis of wild barley using diversity array technology (DArT) and single nucleotide polymorphism (SNP) markers showed that wild barley migrated from Near East Fertile Crescent to Tibetan Plateau, where they have been adapted to the Tibet high altitude and its severe climate (Dai et al. 2012).

Maximising our exploitation of the diversity in wild populations, including materials from diverse climates, will significantly assist breeding and conservation planning (Franks et al. 2007; Hoffmann and Sgro 2011). Despite its importance, relatively little research has focused on how crop wild relatives adapt to their native environments, with much research focusing instead on the model plants *Arabidopsis thaliana* (Weigel and Nordborg 2015) and *Medicago truncatula* (Yoder et al. 2014). The capability of next generation sequencing technologies to generate data for a range of non-model species has transformed our ability to identify the genes underpinning adaptation (Stapley et al. 2010). For example, the adaptation of *Arabidopsis lyrata* to serpentine soils was found at least partly due to gene copy number variants (Turner et al. 2010). One productive avenue of research is to identify genetic variation for climatic adaptation by sampling accessions across environmental gradients such as aridity or temperature (Berger and Ludwig 2014). The analysis may be considered a “reverse ecology” approach for identifying loci that may underlie adaptation to clinal climate variation by genome-wide association with bioclimatic variables as proxy traits for environmental adaptation—without prior knowledge of specific traits mediating that adaptation (Yoder et al. 2014). The rationale of the approach is that if there is genetic adaptation along climatic gradients, then regions of the genome under strong genetic selection for adaptation may be identified. One aim of this study is to test this hypothesis.

The critical need for understanding and conserving crop wild relative diversity motivated Vincent et al. (2013) to develop a prioritised crop wild relative inventory for 1667 taxa. Here, we focus on one of those taxa—narrow-leafed lupin (*Lupinus angustifolius* L.)—as a model to study migration and climatic adaptation in the ancestral wild populations of this species. Narrow-leafed lupin (*Lupinus angustifolius* L.) is an important minor grain legume crop, being grown over 750,000 hectares mainly in Australia, Poland, Russia and Germany (FAO 2014). It has a unique adaptation to infertile, light-textured and acidic soils, where many other crops fail to thrive (Gladstones 1998). As with other members of the *Lupinus* genus, narrow-leafed lupin has the ability for co-adaptation with nitrogen-fixing root symbiont *Bradyrhizobium* sp. and to effectively mobilise soil phosphorus, which makes them of great importance in sustainable farming in rotation with cereals (Lambers et al. 2013; Seymour et al. 2012). Currently, narrow-leafed lupin grain is used mainly for animal feed and in aquaculture but interest is growing in its use in the human diet (e.g. as components in bread, pasta and other products), due to the excellent nutritional properties of the seeds, being gluten-free, high in protein and dietary fibre, and low in starch and fat (Caballero et al. 2015).

Wild narrow-leafed lupin exhibits the winter-annual life-cycle typical of many Mediterranean species. Seeds germinate in the autumn with the onset of rains, establish in rosette form during the winter and then in spring when temperature rises and photoperiod increases, growth increases rapidly and the plants transition to flowering (Kurlovich and Heinanen 2002). Time of flowering of wild lupins is mediated by their responsiveness to vernalisation (cold conditions), day length and ambient temperature (Berger et al. 2012b). We are only now beginning to understand the genetic control of phenology in narrow-leafed lupin. While wild narrow-leafed lupin has a Mediterranean distribution, its adaptation to summer cultivation in northern Europe and winter cultivation in southern Australia was achieved through the removal of the vernalisation response, which was recently shown to be controlled by a mutation in a *Flowering Locus T* (*FT*) gene (Nelson et al. 2017). A recent study in three Old World lupin species, including *L. angustifolius*, *L. albus* and *L. luteus,* demonstrated that phenology in all three has been under strong selection along aridity gradients (Jens Berger, unpublished data). While narrow-leafed lupin domestication was only completed in the twentieth century, there is extensive evidence of its use by Mediterranean cultures for centuries (Gladstones 1970). This is reflected in enlarged seeds on plants growing near agricultural sites (Gladstones and Crosbie 1979) and a wealth of morphology diversity in the Aegean region consistent with extensive movement of seed around that region (Clements and Cowling 1994). The centre of origin of narrow-leafed lupin itself is unknown.

Narrow-leafed lupin cultivars possess a very limited adaptive range relative to their wild ancestors (Berger et al. 2012a). Fortunately, extensive and well-annotated collections of genetic resources of wild narrow-leafed lupin are available (Berger et al. 2013; Wolko et al. 2011). This, together with the recent release of a comprehensive reference genome for narrow-leafed lupin (Hane et al. 2017), provides an excellent opportunity to study regional adaptation in wild narrow-leafed lupin within their full native range. To this end, using different genomics and bioinformatics approaches, we addressed the following questions: (1) How does genetic diversity in narrow-leafed lupin vary across its native range? (2) What can we deduce about the demographic history of this species? and (3) Can we detect regions of the genome associated with climatic adaptation? The information will be valuable to understand the origins of a wild crop ancestor such as narrow-leafed lupin, which serves as a model to identify sources of genetic and adaptive diversity to adapt crops to changing environments.

## Materials and methods

### Plant materials and climatic variables

A total of 142 wild accessions of narrow-leafed lupin from 11 countries across the Mediterranean basin were obtained from the Australian Lupin Collection, Department of Agriculture and Food Western Australia (DAFWA; Online Resource 1). This subset of 142 accessions were selected from 1248 wild accessions characterised by Berger et al. (2012a) using 137 array-based DArT markers, and encompassed the maximum genetic and phenotypic diversity from across the geographic range for this species (Gladstones 1998). Phenotypic data including alkaloid status, pod dehiscence, physical dormancy (hard versus soft seededness), rain, soil pH at collection site, flower colour, time to flowering from sowing date (flowering time), height at maturity and 100 seed weight were from a study of Gladstones and Crosbie (1979). Additional passport information was kindly provided by the Australian Lupin Collection, including the geographic coordinates of accessions from geo-referencing of collection site data. Geographic coordinates for each accession were used to extract 19 climatic variables drawn from the years 1960–1990 from WorldClim (http://www.worldclim.org/bioclim) (Hijmans et al. 2005; WorldClim 2006), using ArcMap GIS (Geographic Information System) software 10.3.1 version (Esri, CA, USA). Information on geographic, phenotypic and WorldClim variables are provided in Online Resource 1.

### DArTseq genotyping

DNA was extracted from leaves of single plants from each accession using a standard CTAB method (Doyle and Doyle 1990). The quality and quantity of extracted DNA were assessed using standard agarose electrophoresis and Qubit™ assays (http://www.Invitrogen.com/qubit).

The DNA concentration of each sample was adjusted to 20 ng/μL and subjected to DArTSeq™ (hereafter, DArTseq) genotyping at Diversity Arrays Technology Pty Ltd, Canberra, Australia (Sansaloni et al. 2011). DNA samples are processed in digestion/ligation reactions principally as per Kilian et al. (2012), but replacing a single *Pst*I-compatible adaptor with two different adaptors corresponding to two different Restriction Enzyme overhangs. The *Pst*I-compatible adapter was designed to include Illumina flowcell attachment sequence, sequencing primer sequence and staggered, varying length barcode region, similar to the sequence reported by Elshire et al. (2011). Reverse adapter contained flowcell attachment region and *Mse*I-compatible overhang sequence.

Only “mixed fragments” (*Pst*I-*Hpa*II) were effectively amplified in 30 rounds of PCR using the following reaction conditions: 94 °C for 1 min, then 30 cycles of 94 °C for 20 s, 58 °C for 30 s and 72 °C for 45 s, finishing with 72 °C for 7 min. After, PCR equimolar amounts of amplification products from each sample of the 96-well microtiter plate were bulked and applied to c-Bot (Illumina) bridge PCR followed by sequencing on Illumina Hiseq2500. The sequencing (single read) was run for 77 cycles.

Sequences generated from each lane were processed using proprietary DArT analytical pipelines. In the primary pipeline, the fastq files were first processed to filtre away poor quality sequences, applying more stringent selection criteria to the barcode region compared to the rest of the sequence. Approximately 2,500,000 (± 7%) sequences per barcode/sample were used in marker calling. Finally, identical sequences were collapsed into “fastqcall” files. These files were used in the secondary pipeline for DArT PL’s proprietary SNP and SilicoDArT (presence/absence of restriction fragments in representation) calling algorithms (DArTsoft14). Co-dominant SNP markers were the focus of the study, as they are able to distinguish heterozygous and homozygous loci, which was necessary for downstream analyses, and were filtered using different thresholds for various analyses. For demography analyses, SNP markers which had positions mapped to pseudo-chromosomes were used. For phylogenetic, population structure, linkage disequilibrium and association studies, SNPs with more than ≥ 25% missing data or ≥ 12.8% heterozygosity were eliminated. DArTseq reads were aligned to the *L. angustifolius* cv. Tanjil reference genome (Hane et al. 2017) using NUCmer aligner distributed with MUMmer (Delcher et al. 1999), with setting the minimum cluster length parameter (-c flag) to 25 bp, and allowing ≤ 3 matches. Then, the position of SNPs relative to Tanjil reference genome was determined from SNP positions on the DArTseq reads and the reads’ match position on the reference, summarised in Variant Call Format (VCF) relative to the lupin reference genome (Hane et al. 2017). The VCF file was validated using the Genome Analysis Toolkit (GATK) (McKenna et al. 2010).

### Assessing population structure and climate adaptation across the Mediterranean basin

Two different methods were employed to identify population structure: Principal Component Analysis (PCA) was performed using EIGENSTRAT to assess genetic diversity and to correct for population stratification (Price et al. 2006). We also used fastSTRUCTURE at *K* = 2 to *K* = 12, using default parameters (Raj et al. 2014). The estimation of optimum *K* was obtained using the algorithm implemented in fastSTRUCTURE to choose model complexity (Raj et al. 2014). To understand the relationship between phylogeny and geographical distribution, we used GenGIS 2.4.0 (Parks et al. 2013). The unrooted distance-based phylogeny tree, which was implemented as Newick format in GenGIS, was obtained using Neighbor Joining method within the NEIGHBOR package in PHYLIP (Felsenstein 1989).

A Pearson correlation coefficient heat-map was calculated using the heat-map function in R version 3.3.0 (R Core Team 2016) for all phenotypic characteristics and climate data extracted from WorldClim.

To test the association between the SNP markers and the WorldClim variables which were significantly correlated with important agronomic traits such as flowering time—referred to hereafter as climatic variables—we carried out genome-wide association studies (GWAS) using GAPIT (Lipka et al. 2012) with the first two principal components as covariates, for a dominant model and a minor allele frequency (MAF) cutoff of 0.01.

Next, SNPs that were highly associated with our traits of interest were analysed using SnpEff version 4.3 (Cingolani et al. 2012), which annotates genetic variants and predicts their effects on genes. SnpEff annotations were compared with the predicted proteins reported by Hane et al. (2017).

GenStat version 16.2 (VSN International 2011) was used to perform analysis of variance (ANOVA) among groups of our accessions (East/West Mediterranean) which were inferred from GenGIS phylogeny result. This was undertaken to estimate the significance of variation between phenotypic traits and climatic variables of different samples belonging to separate geographic groups.

Linkage disequilibrium (LD) as measured by *r*^2^ was calculated for all values (–ld-window-*r*^2^ 0) for every SNP within a window of 1 Mb using Plink (Purcell et al. 2007). The mean *r*^2^ values pooled over all 20 chromosomes for western and eastern wild germplasm were calculated and plotted using R v3.3.0 (R Core Team 2016). Analysis of haplotype blocks was performed using Haploview (Barrett et al. 2005).

### Demographic analyses

To infer the demographic history of the wild populations of *L. angustifolius,* we used the models implemented in the software dadi (Gutenkunst et al. 2009). This method uses the site frequency spectrum (SFS) of populations, i.e. the distribution of allele frequencies across SNPs, to infer the demographic history of populations. The observed SFS was compared to the expected SFS under different demographic models, and using Maximum Likelihood, estimated both the likelihood of the different models and their parameters values.

For SFS analysis, we included all 38,948 SNPs with known position in the pseudo-chromosomes of the *L. angustifolius* cv. Tanjil genome assembly (Hane et al. 2017). Based on the fastSTRUCTURE and phylogeny results, we defined three wild populations for this analysis: the western population consisting of samples from Morocco, Portugal and Spain (*n* = 74); the central population containing samples from France and Italy (*n* = 21); and the eastern population containing the samples from Greece, Cyprus and Turkey (*n* = 45). As the ancestral state of each SNP is uncertain, we folded the SFS in dadi before fitting the Isolation with Migration (IM) model. Preliminary analysis suggested that singletons, i.e. alleles found only once in the population, were under-represented in all populations, suggesting a bias in the SNP calling protocol. To avoid biasing the demographic inferences due to this, we excluded all singletons from the calculations of the SFS.

We performed two types of analyses. First, we investigated the demographic history of each population separately, by fitting the default single population models available in dadi (Table 1). We then investigated the Isolation with Migration (IM) model, which compares pairs of populations and estimates their relative size, time of divergence, the population size changes for each population, and the amount of gene flow in each direction (Table 1). To ensure convergence of the parameter estimates, for each model investigated we performed 20 runs with random starting values for each parameter and large search regions. After analysis of these results, we defined smaller regions of parameter space with high likelihood and performed a second round of 20 searches from random starting points within this region.

**Table 1:** Description and parameters considered in each demographic model, implemented in dadi

Model type^a^	Model name	Description	Parameters estimated^b^
1D	Neutral	Constant population size	None
1D	Two epoch	Instantaneous population size change	nu: current population size; *T*: time of population size change
1D	Growth	Exponential population growth or decline	nu: current population size; *T*: time of start of population growth or decline
1D	Bottle Growth	Instantaneous population size change followed by exponential growth or decline.	nuB: population size after instantaneous change; nuF: current population size; *T*: time of instantaneous population size change and growth/decline start
1D	Three epoch	Two instantaneous population size changes (i.e. 3 periods of constant population size)	nuB, nuF: population sizes of second and third epochs. TB: duration of second epoch; TF: duration of third epoch
2D	Isolation with migration	Two populations split model with unequal sizes, independent size changes and migration values in both directions	s: population size of first population after split (population 2 has size 1-s); nu1, nu2: current population sizes of the two populations; T: time of population split; M12: migration from population 2 to population 1; M21: migration from population 1 to population 2

^a^One-dimensional models (1D) consider a single population, while two-dimensional (2D) models consider two populations at a time

^b^All population sizes are relative to the size of the ancestral population before any changes or splits occur (Na). All time parameters are in units of 2 × Na. Migration values are expressed in units of 2 × Na × *m*, where *m* is the proportion of the receiving population made of immigrants in each generation

## Results

DArTseq analysis generated 45,230 co-dominant SNPs markers in 142 wild narrow-leafed lupin accessions. For the demography analyses, we used a subset of SNPs that mapped to pseudo-chromosomes which included 38,948 SNPs. For other analyses, loci with excessive missing and heterozygote values were removed (16,714 SNPs remained). Due to the evidence of whole-genome triplication in the genome of narrow-leafed lupin (Hane et al. 2017; Kroc et al. 2014), we kept those SNPs that had a maximum of three matches in the reference genome (11,690 SNPs remained). Consequently, these 11,690 SNPs were utilised for phylogenetic, population structure, linkage disequilibrium and association studies. The allelic profile per accession for these 45,230 and 11,690 SNP sets are reported in the Online Resource 2 and 3, respectively.

### Population structure

**Fig. 1:**
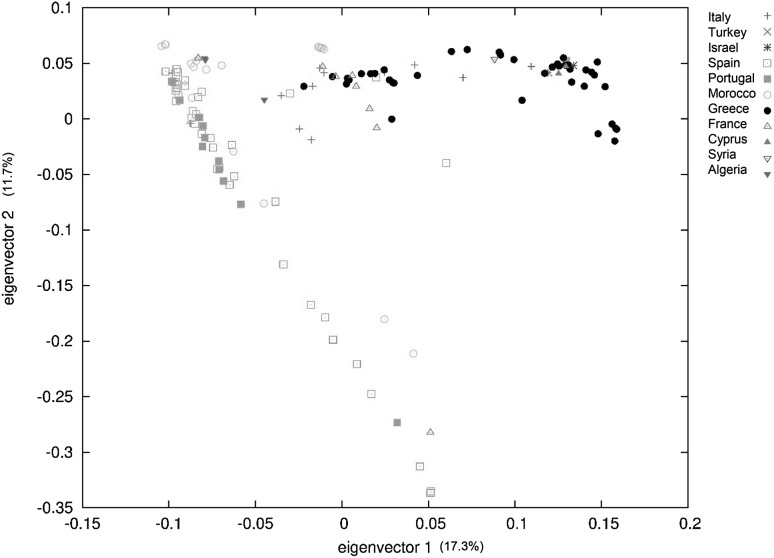
Principal component analysis (PCA) for 142 accessions of wild narrow-leafed lupin labelled by country of origin

Principal component analysis (PCA) depicted a vivid division between East and West Mediterranean accessions (Fig. 1). The first and second principal components (PCs), together accounted for 29% of the genetic variation, and despite a few exceptions (mainly from Italy and France), it differentiated the genotypes into two groups, western and eastern Mediterranean comprising 97 and 45 accessions, respectively (Online Resource 4).

**Fig. 2:**
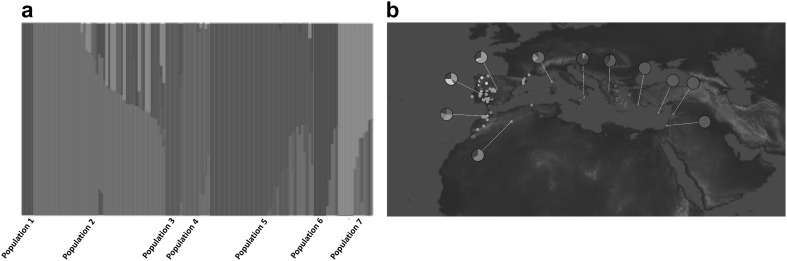
Population stratification among 142 accessions of wild narrow-leafed lupin (*K* = 7) using fastSTRUCTURE. Each colour denotes a population affiliation. **a** Seven population groups identified among 142 wild narrow-leafed lupin accessions. **b** GenGIS plot showing geographic distribution of seven fastSTRUCTURE groups. Dots represent collection sites of accessions colour-coded by fastSTRUCTURE group (*K* = 7), with grey shading denoting admixed accessions. Pie charts represent the frequencies of populations within each country using the same colour coding (colour figure online)

The second method we used to probe the relationships among accessions was the Bayesian clustering algorithm applied in fastSTRUCTURE programme (Raj et al. 2014). To reveal population structure, we investigated different numbers of populations from 2 to 12 (*K*2–12) (Online Resource 5). The internal algorithm in fastSTRUCTURE for multiple choices of *K* determined that population numbers *K* = 7 or *K* = 8 best explained the variation in the dataset. To determine which one of these two models (*K* = 7 or *K* = 8) better fitted the data, we subjected both models to phylogenetic analysis. Again, as both models appeared plausible, the simpler model (*K* = 7) was selected (Fig. 2a, b). We used a frequency threshold of > 0.7 to assign accessions to their corresponding populations. Where accessions had population affinity values below 0.7, they were categorised as Admixed. Structure grouping depicted seven populations: one population for the entire eastern Mediterranean region (Population 5) and the remaining six populations for the western Mediterranean region indicating greater stratification centred on the Iberian Peninsula. Admixed populations were distributed across the Mediterranean but were particularly frequent at the intersection of East and West (centred on Italy).

### Phylogenetic analyses

**Fig. 3:**
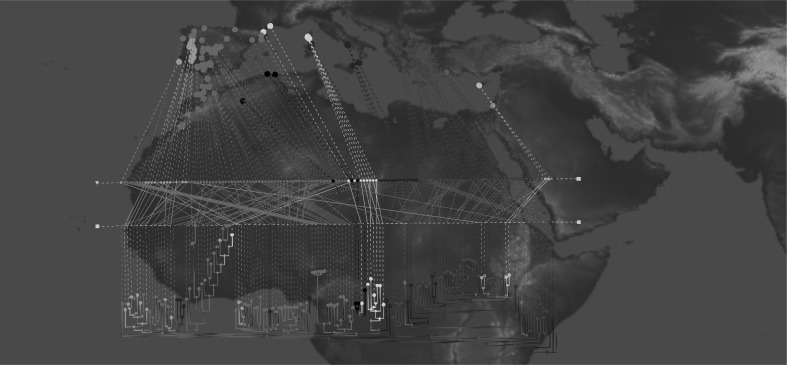
Phylogenetic analysis of 142 accessions of wild narrow-leafed lupin using GenGIS. Accessions are colour coded based on their country of origin, as following: *orange* Israel, *dark green* Syria, *turquoise blue* Cyprus, *sky blue* Turkey, *purple* Greece, *dark blue* Italy, *pink* France, *red* Spain, *yellow* Portugal, *light green* Morocco, *black* Algeria (colour figure online)

Phylogenetic relationships were captured within an unrooted tree based on distances calculated from 11,690 DArTseq SNP markers. This shows a distinct East/West Mediterranean division, with most accessions from Morocco, Algeria, Spain, Portugal and France clustering together as a western group, while the remaining accessions mainly clustered together as an eastern group (Fig. 3). This delineation into East/West groups was generally congruent with the PCA and fastSTRUCTURE-defined groupings (Online Resource 4).

The phylogenetic grouping of most accessions corresponded to their geographical locations with the exception of three accessions from Spain and Portugal which were clustered within the eastern group (accessions P22666, P28221 and P26423), and seven accessions from Italy and one accession from Greece that fell within the western clusters (Accessions P20720, P20724, P25040, P25051, P25052, P26107, P26109 and P26991).

### Investigating historic population bottlenecks

**Fig. 4:**
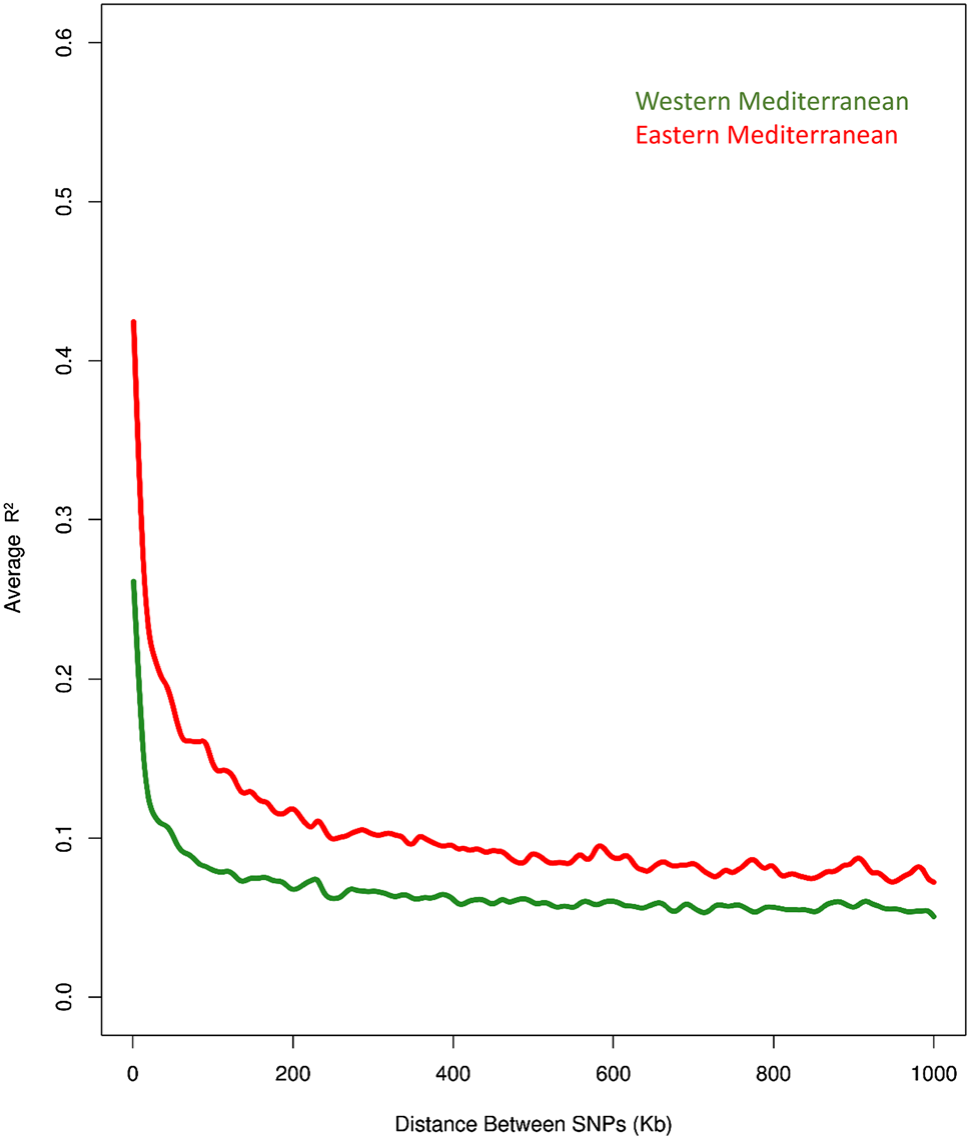
Decay of genome-wide linkage disequilibrium in 142 wild narrow-leafed lupin accessions for western and eastern populations

To explore further how genomic diversity differs between the distinct eastern and western Mediterranean population groups and to infer the historic levels of genetic diversity in each population group, we investigated linkage disequilibrium (LD) in these two population groups and conducted demographic analyses. The western population group showed far lower average maximum LD (0.2) than the eastern population group (0.4), and the LD decayed more rapidly in the western populations (Fig. 4). We investigated population bottleneck further through demographic analyses.

### Demography analyses

The different single population models investigated returned very consistent results, with the western population group showing relatively small population size changes and an older age, while the eastern population group appearing much younger, having experienced a recent and severe bottleneck, and to have subsequently increased its population size considerably (Table 2). There was also evidence that a small central group (comprising accessions identified as admixed in the fastSTRUCTURE analysis) was distinguishable from western and eastern groups. Like the eastern population group, the central population group showed evidence of a recent and severe bottleneck followed by population increase.

**Table 2:** Maximum Likelihood parameter estimates, and log-likelihood (LL), of the single population demographic models considered

Model	Parameters estimated	Western population	Central population	Eastern population
Neutral	None	LL = − 225.598	LL = − 133.27	LL = − 126.763
Two epoch	nu: current population size; *T*: time of population size change	nu = 3.189; *T* = 5.57 LL = − 183.256	nu = 0.305; *T* = 0.7 LL = − 73.878	nu = 0.76; *T* = 0.2 LL = − 89.24
Growth	nu: current population size; *T*: time of start of population growth or decline	nu = 2.27; *T* = 10.9 LL = − 190.365	NA^a^	nu = 0.72; *T* = 0.47 LL = − 89.589
Bottle Growth	nuB: population size after instantaneous change; nuF: current population size; *T*: time of instantaneous population size change and growth/decline start	nuB = 9.8; nuF = 1.97 *T* = 2.4; LL = − 151.068	nuB = 0.1; nuF = 10.54 *T* = 0.259; LL = − 46.934	nuB = 0.18; nuF = 59.9 *T* = 0.116; LL = − 83.393
Three epoch	nuB, nuF: population sizes of second and third epochs. TB: duration of second epoch; TF: duration of third epoch	nuB = 14.10; nuF = 2.84 TB = 2.33; TF = 1.13; LL = − 145.814	nuB = 0.05; nuF = 14.01 TB = 0.17; TF = 0.05; LL = − 46.50	nuB = 0.04; nuF = 78.03 TB = 0.004; TF = 0.172; LL = − 81.747

For more details of model information and parameters’ description, please see Table 1

^a^The Growth model for the central population did not converge

We then considered the Isolation with Migration (IM) model with each pair of populations. The IM model had six free parameters: the relative size of the two populations after they split, time of the split, modern population sizes of each population and effective migration rates in two directions. We found that population split sizes were highly unequal, with each pairwise comparison suggesting that the eastern population emerged from a very small proportion of the ancestral population (*s* = 0.95–0.99, Table 3). The extent of migration from West to East was much higher than in the reverse direction (*m*12 = 0.06–1.13 while *m*21 = 9.9–15.11, Table 3).

**Table 3:** Maximum likelihood parameter estimates of the Isolation with Migration model, for each population pair analysed

Population 1	Population 2	*S*	nu1	nu2	*T*	*m*12	*m*21
West	Central	0.95	0.6769	1.811	0.0652	0.9915	7.4456
West	East	0.9959	0.215	0.919	0.0235	0.0687	15.116
Central	East	0.988	2.49	0.55	0.069	1.133	9.9

*S* population size of first population after split (population 2 has size 1-s), *nu1* current size of population 1, *nu2* current size of population 2, *T* time of population split, *m12* migration from population 2 to population 1, *m21* migration from population 1 to population 2

### Investigating adaptation of wild narrow-leafed lupin in West/East Mediterranean

**Fig. 5:**
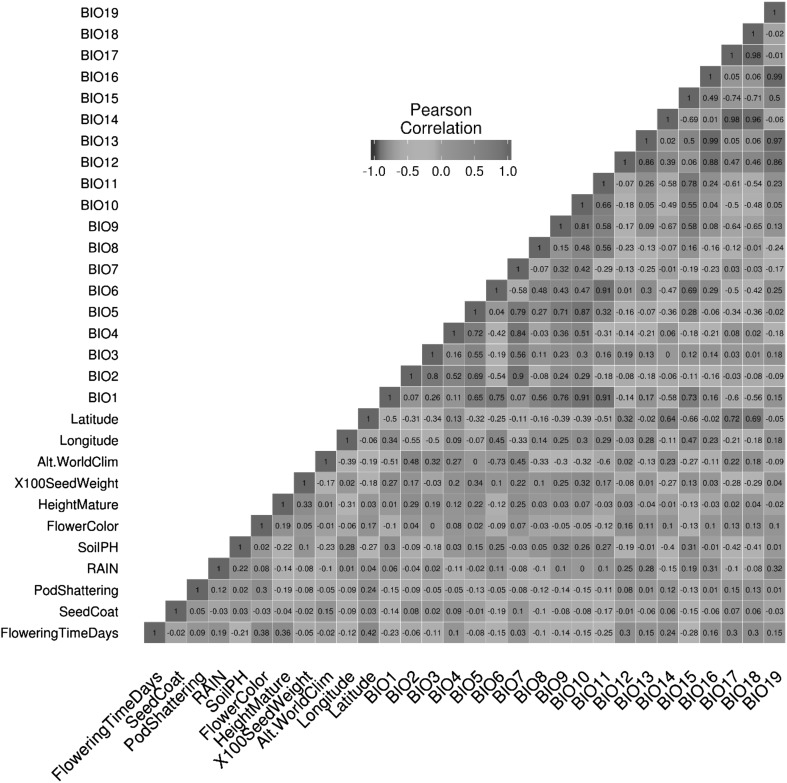
Pearson correlation heat-map of climatic and geographic variables with phenotypic traits for 142 narrow-leafed lupin accessions from across the Mediterranean Basin. WorldClim data abbreviations are as follows: *BIO1* annual mean temperature, *BIO2* mean diurnal range [mean of monthly (max temp − min temp)]; *BIO3* isothermality (BIO2/BIO7) (× 100), *BIO4* temperature seasonality (standard deviation × 100), *BIO5* max temperature of warmest month, *BIO6* min temperature of coldest month, *BIO7* temperature annual range (BIO5–BIO6), *BIO8* mean temperature of wettest quarter, *BIO9* mean temperature of driest quarter, *BIO10* mean temperature of warmest quarter, *BIO11* mean temperature of coldest quarter, *BIO12* annual precipitation, *BIO13* precipitation of wettest month, *BIO14* precipitation of driest month, *BIO15* precipitation seasonality (coefficient of variation), *BIO16* precipitation of wettest quarter, *BIO17* precipitation of driest quarter, *BIO18* precipitation of warmest quarter, *BIO19* precipitation of coldest quarter. Temperatures are in degrees Celsius × 10 and precipitation in millimetres

We investigated adaptation parameters distinguishing West and East populations. Climatic variables from the sites of origin of each accession (extracted from the WorldClim database) were correlated with phenotypic traits (number of days to flowering, hard/soft seededness, pod dehiscence status, height at maturity and 100-seed weight). As separate correlation analyses for Western and Eastern populations showed similar trends, we only present one correlation matrix for both West and East together (Fig. 5). Variation in flowering time was significantly (at *p* < 0.01 and degree of freedom of 140) associated with some climatic/eco-geographic variables, including precipitation in different months/quarters (BIO14 and BIO17, respectively), latitude and annual mean temperature (BIO1). Positive correlations were observed between flowering time and latitude, and between flowering time and precipitation in the driest month and driest quarter, while the correlation between flowering time and annual mean temperature was negative.

### Exploring phenotypic variation corresponding to geographical location

We next sought to determine how geographical location (East or West Mediterranean, inferred from phylogenetic tree) could explain observed phenotypic variation. In total, 53 and 89 accessions were labelled as eastern and western groups, respectively (Online Resource 1). A one-way analysis of variance (ANOVA) indicated there was significant (*p* < 0.05) variation in phenotypic traits (such as flowering time) and climatic variables (e.g. Precipitation of driest month/quarter, etc.) between samples from West/East Mediterranean. Details of significant variation traits are provided in Table 4. It was clear that flowering time differed between West (late flowering) and East (early flowering) populations (*p* < 0.05 Table 4) and that flowering time was significantly correlated with some climatic variables such as precipitation of driest month/quarter, etc. Based on this, we next looked for regions of the narrow-leafed lupin genome that appear to be associated with adaptation.

**Table 4:** Summary of significant variation in phenotypic and WorldClim data between western and eastern Mediterranean populations of wild narrow-leafed lupin as inferred by one-way ANOVA analysis

WorldClim/phenotypes	Mean Western population	Mean Eastern population	*F* pr.
Altitude (m above sea level)	545.240	338.000	< 0.001
BIO1: annual mean temperature (°C × 10)	145.943	160.264	< 0.001
BIO2: mean diurnal range [mean of monthly (max temp − min temp)] (°C × 10)	103.693	80.208	< 0.001
BIO3: isothermality (BIO2/BIO7) (× 10)	37.977	32.566	< 0.001
BIO6: min temperature of coldest month (°C × 1010)	29.602	54.333	< 0.001
BIO7: temperature annual range (°C × 1010)	268.193	240.528	< 0.001
BIO9: mean temperature of driest quarter (°C × 1010)	211.875	234.698	< 0.001
BIO10: mean temperature of warmest quarter (°C × 1010)	221.227	235.604	< 0.001
BIO11: mean temperature of coldest quarter (°C × 1010)	76.932	89.491	0.004
BIO13: precipitation of wettest month (mm)	99.170	118.528	0.011
BIO14: precipitation of driest month (mm)	11.557	7.000	0.024
BIO15: precipitation seasonality (mm)	50.580	70.340	< 0.001
BIO16: precipitation of wettest quarter (mm)	273.190	314.400	0.046
BIO17: precipitation of driest quarter (mm)	55.591	30.585	< 0.001
BIO18: precipitation of warmest quarter (mm)	64.227	34.887	< 0.001
Flowering time (days)	106.403	102.269	0.011
Height at maturity (cm)	91.033	80.081	0.002
Rain (mm)	771.480	601.650	< 0.001

### Identifying regions of the genome associated with adaptation

**Fig. 6:**
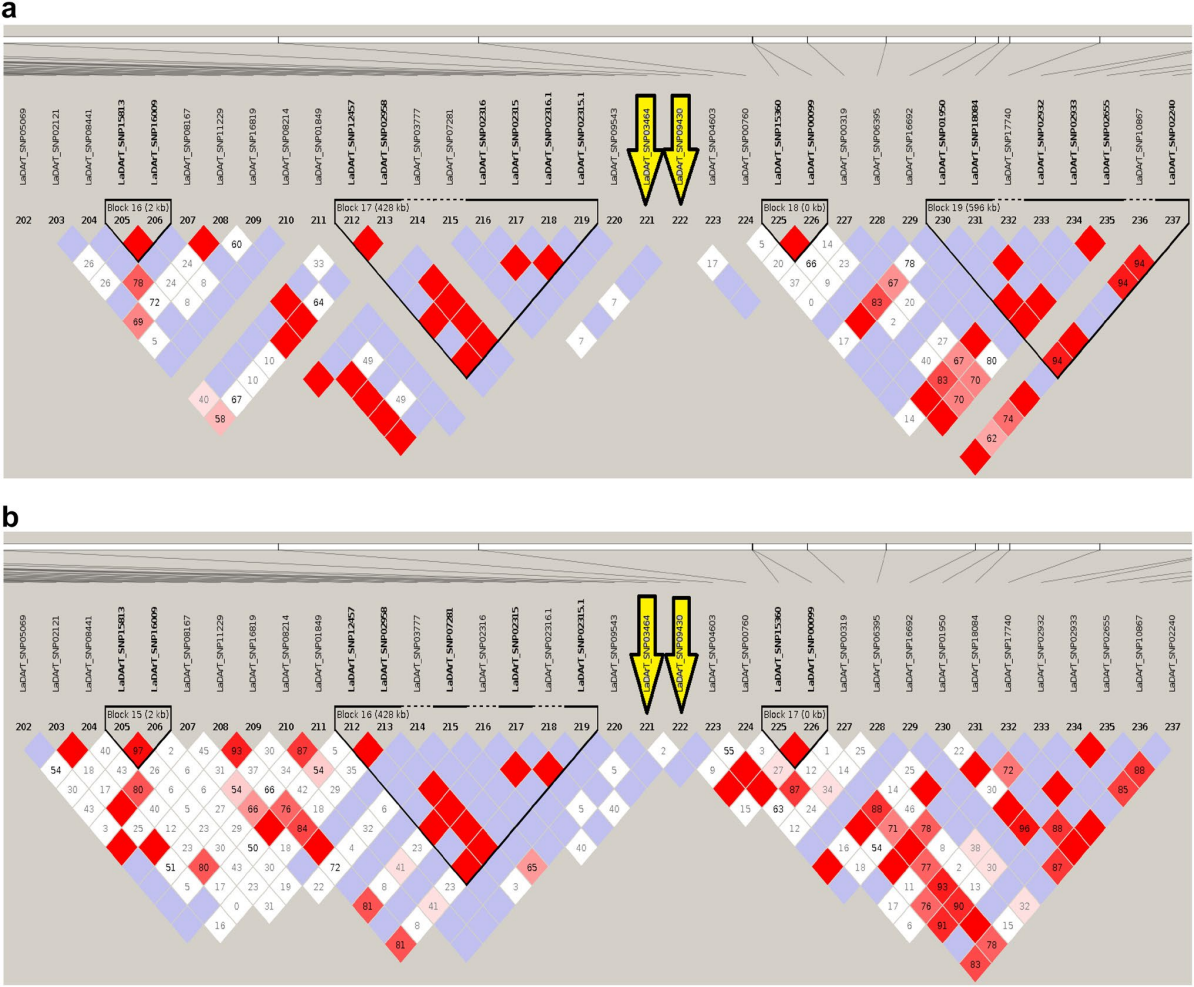
Comparison of haplotype blocks between East (**a**) and West (**b**) Mediterranean accessions of wild narrow-leafed lupin on chromosome NLL-10 in the *LanFTc1* gene region (flanked by markers indicated with yellow arrows). Each colourful diamond shows correlation between two markers. The extent of correlation between markers is shown by the shade of colours, with white denoting recombination and red denoting strong linkage disequilibrium. Purple and grey diamonds indicate uninformative and monomorphic marker comparisons, respectively. Black triangles show the haplotype blocks (colour figure online)

First, we focused in more detail on the distribution of LD across the genome. Vernalisation is a key driver of flowering time in narrow-leafed lupin, which is controlled by the *FT* homologue *LanFTc1* at the *Ku* locus in domesticated varieties (Nelson et al. 2017). We, therefore, compared the haplotype block of East (Fig. 6a) and West (Fig. 6b) populations in the *LanFTc1* gene region on chromosome NLL-10. Although the overall pattern of haplotype blocks in the *LanFTc1* region was similar between West/East populations, the comparison revealed evidence of greater historical recombination within western Mediterranean population compared to East (Fig. 6a, b).

**Fig. 7:**
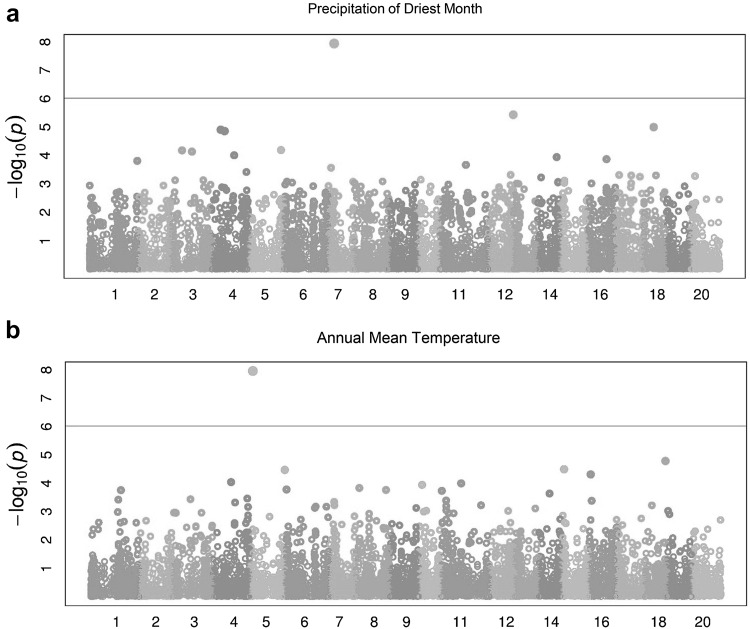
Manhattan plot of genome-wide association study (GWAS) using 11,690 SNPs markers for precipitation of driest month/quarter (mm) (**a**) and annual mean temperature (°C × 10) (**b**) on 142 wild narrow-leafed lupin accessions. The *X*-axis represents physical distance along the 20 narrow-leafed lupin chromosomes, NLL-01 to NLL-20. SNPs above the threshold line [green line; − log10(*p*) = 6] are highly significantly associated with precipitation of driest month and annual mean temperature, respectively (colour figure online)

Second, we investigated association between markers and phenotypic traits (i.e. flowering time, flower colour, hard/soft seededness, alkaloid status, pod dehiscence, height at maturity and 100 seed weight), as well as association between markers and climatic variables. Although GWAS analyses did not highlight any significant association between markers and phenotypic traits, it pointed out a strong association with two of the climatic variables. A SNP on position 4,582,844 bp of chromosome NLL-07 (LaDArT_SNP18940) was found to be strongly associated (*p* = 1.143e−08) with precipitation of driest month/quarter and LaDArT_SNP09086 locus on chromosome NLL-05 (position: 2,350,287 bp) was significantly associated (*p* = 1.089e−08) with annual mean temperature (Fig. 7). The genic composition of these genomic regions was then investigated. Taking a conservative approach, we considered the entire regions flanked by non-associated SNP markers as potentially containing gene candidates. This revealed 31 genes in an interval spanning 240 Kb on chromosome NLL-07 around the SNP associated with precipitation of driest month/quarter and 5 genes in an interval spanning 47 Kb on NLL-05 around the SNP associated with annual mean temperature. These genes and their functional annotations are listed in Online Resource 6. No standout candidates (such as flowering time genes) based on functional annotations were identified.

## Discussion

### Distribution pattern and migration of narrow-leafed lupin

This is the first pan-Mediterranean analysis of genetic diversity in wild narrow-leafed lupin. We observed a strong East–West division in wild narrow-leafed lupin, an eastward migration, and greater population structure in the western Mediterranean.

Migration of plant populations is influenced by a large number of factors. The complex geological history of the Mediterranean region along with climate fluctuations enforce plant migration, strong selection pressure on plant traits and shifts in plants species’ distribution. These two natural phenomena accompanied by the impact of human activities have shaped plant variation and evolution in the Mediterranean region (Thompson 2005). Thompson (2005) described three main poles of plant diversity in the Mediterranean region, which include (1) the Iberian Peninsula, (2) the Balkans and Aegean, and (3) Anatolia and Cyprus. At a wider genus level, Gladstones (1998) postulated western Asia (Turkey, Syria and nearby area) as the probable origin of smooth-seeded Mediterranean lupins, including narrow-leafed lupin, and that they have spread westwards along Mediterranean. Early phenotypic studies suggested the Aegean region as the centre of diversity of narrow-leafed lupin (Clements and Cowling 1994), with significant distribution of wild types in North Africa and Iberia (Gladstones 1998). The results of our demographic analysis strongly suggest that narrow-leafed lupin originated in the western part of its current distribution range, and that range expansion proceeded eastwards and was accompanied by strong founder effects (Table 2). The current populations seem to be still exchanging large amount of migrants, particularly from western towards eastern populations (Table 3). There is substantial admixture in the central region between West and East, centred on Italy and France (Fig. 2).

At present, it is not possible to estimate absolute ages for separation between western and eastern Mediterranean narrow-leafed lupin. Our results are consistent with east–west vicariance, one of the two common distribution patterns of higher plants in Mediterranean basin, which is attributed to population isolation in association with climatic change and human immigration as described by Thompson (2005). One well-known example of east–west vicariance is Mediterranean oak, in which extremes of morphological diversity were observed in a single variable complex (Barbero et al. 1992). It is assumed that the two taxa belonging to western Mediterranean oak split up due to the effect of glaciation during the refuge period. Owing to its particular geography, the Iberian Peninsula hosted multiple glacial refugia, which were “isolated from one another by the harsh climate of the high central Iberian plateau” (Gómez and Lunt 2007 p. 156). A similar population history could explain the greater population structure observed in western Mediterranean wild narrow-leafed lupins. Interestingly, great morphological diversity is also reported in lupins of eastern Mediterranean (Clements and Cowling 1994) despite the lower genetic diversity observed in that group in this present study (Figs. 1, 2, 4). This could be due to the fact that centre of earlier civilization was in Eastern Mediterranean, and as a result the semi-wild populations of narrow-leafed lupin in that region may have undergone more human selection and physical relocation.

Consistent with demography (Table 2) and population structure results (Fig. 2), the observed higher extent of LD in the eastern population compared to the western population (Fig. 4) could be an indicator of severe founder effect that gave rise to this population, whereby a small number of ancestral haplotypes from the western Mediterranean contributed to the emergence of today’s eastern population haplotypes. The lower extent of LD in the western population is an indicator of more recombination events which broke down the ancestral haplotypes of this population. This higher rate of recombination could be due to the older age, larger population size and greater diversity.

Recognising Iberian Peninsula as a variability hotspot for the wild ancestral populations of narrow-leafed lupin will help in planning to collect future genetic resources efficiently. The similar approach could be applied to other crop ancestral populations to assist their conservation plans and ensure the genetic resources are exploited effectively.

### Selection for climatic adaptation

Adaptation is a continuous dynamic process which helps species to keep up with constantly changing environments. The strong seasonal climate of the Mediterranean basin, in particular the association of warm seasons with an effective drought which restrict plant growth, has had a fundamental impact on plant evolution in the region (Thompson 2005), including lupin species as studied by Berger et al. (2017).

Clements and Cowling (1994) study of wild narrow-leafed lupin from the Aegean region showed a negative correlation between flowering times with winter temperature, but positive correlation with the rainfall of the site. As revealed by ANOVA analysis in our study, the western Mediterranean has higher precipitation over the driest month/quarter and a lower annual mean temperature and is late flowering, in comparison with the eastern Mediterranean (Table 4). Shifts in phenology in response to climate change has been reported in many species, such as shifts in flowering time of *Brassica rapa* in response to drought (Franks et al. 2007; Parmesan and Yohe 2003; Pulido and Berthold 2010).

Flowering time is a key adaptation trait, which is affected by the interaction of genetic factors with environmental cues. Following up with the significant variation between flowering time of western and eastern population, we compared the haplotype block surrounding the main flowering time gene *LanFTc1* between these two populations. This revealed evidence of recombination in the 571 Kbp region flanking *LanFTc1* within the western population, while that region is monomorphic in the eastern population (Fig. 6a, b). This differentiation in flowering phenology could be explained in light of the migration story of narrow-leafed lupin from West to East. Although irregular in pattern, a West/East bipolar climate has ruled over the Mediterranean basin for at least the last 1000 years (Roberts et al. 2012). To effectively deal with climatic changes resulted from this migration, it appears that selection opted for opportunistic adaptation to the warmer and drier climate of the eastern Mediterranean. Hence, earlier flowering in the eastern Mediterranean population help plants escape drought and heat. This selection may have predated the first use of narrow-leafed lupin by humans and certainly before its domestication was completed in the twentieth Century. So, while early flowering is one of the main selection targets of current lupin breeding, it seems that this trait may have already been under natural selection prior to any domestication event.

### SNP markers associated with climatic variables

Applying trait association analysis to the climatic variables inferred from WorldClim climatic factors highlighted two SNP markers associated with precipitation over the driest month/quarter and annual mean temperature (Fig. 7a, b). There were no clear gene candidates in the respective genomic regions of chromosomes NLL-07 and NLL-05, respectively, based on their functional annotations (Online Resource 6). However, it should be emphasised that the resolution afforded by DArTseq genotyping (approximately one SNP per 13 Kbp of assembled genome) in a sample size of 142 accessions is rather low. Nevertheless, these genomic regions provide starting points for follow-on association studies where we are phenotyping an enlarged set of accessions for flowering time and vernalisation response to identify genes associated with environmental adaptation of narrow-leafed lupin. We are also increasingly using whole-genome resequencing to increase the density of marker genotyping in such studies. A genome–environment association study in 1943 georeferenced sorghum landraces using 404,627 SNPs found signatures of local adaptation at two genes. *Maturity1*, controlling photoperiod sensitivity, was strongly associated with the minimum temperature of the coldest month and *Tannin1*, controlling grain tannins, with mean temperature of the warmest quarter (Lasky et al. 2015).

### Summary conclusion and future directions

For the first time, this study unravelled the diversity and migration history of wild narrow-leafed lupin in the Mediterranean region. This information can be used to help genetic resource managers target regions for sampling wild diversity and to help plant breeders harness genetic diversity in their breeding programmes. Our genome-wide association study between SNP markers and climatic variables could serve as an initial foray into a potential application of combining genetic and spatial datasets. Finally, our comprehensive analytical approach could be applied to understand better other ancestral populations to help build adaptability and reliance into major and minor crops alike.

#### **Author contribution statement**

MMD, MNN and WE conceived the design of this study with input on analytical approaches by DE, PEB and JKH. AK, LGK, KBS and JDB contributed to the DArTseq genotyping. BN and DF conducted the demography analyses. MMD, JKH and PEB identified SNP positions related to narrow-leafed reference genome. BH and PEB provided MMD guidance on coding. MMD and WE performed ANOVA analyses. MMD conducted all other analyses. MMD and MNN led the manuscript preparation. All authors have read and approved the final manuscript.

## Electronic supplementary material

Below is the link to the electronic supplementary material.

Supplementary material 1 (XLSX 38 kb)

Supplementary material 2 (XLSX 24600 kb)

Supplementary material 3 (XLSX 6624 kb)

Principal Component Analysis (PCA) for 142 accessions of wild narrow-leafed lupin colour-coded by phylogeny results (east/west Mediterranean) (PPTX 214 kb)

Supplementary material 5 (PPTX 220 kb)

Supplementary material 6 (XLSX 11 kb)
