# Supplementary figures and images for: Exploring the genetic and adaptive diversity of a pan-Mediterranean crop wild relative: narrow-leafed lupin
Source: Theor Appl Genet. 2018 Jan 20;131(4):887–901. doi: 10.1007/s00122-017-3045-7 (PMC5852200; doi:10.1007/s00122-017-3045-7)

## Slide 1
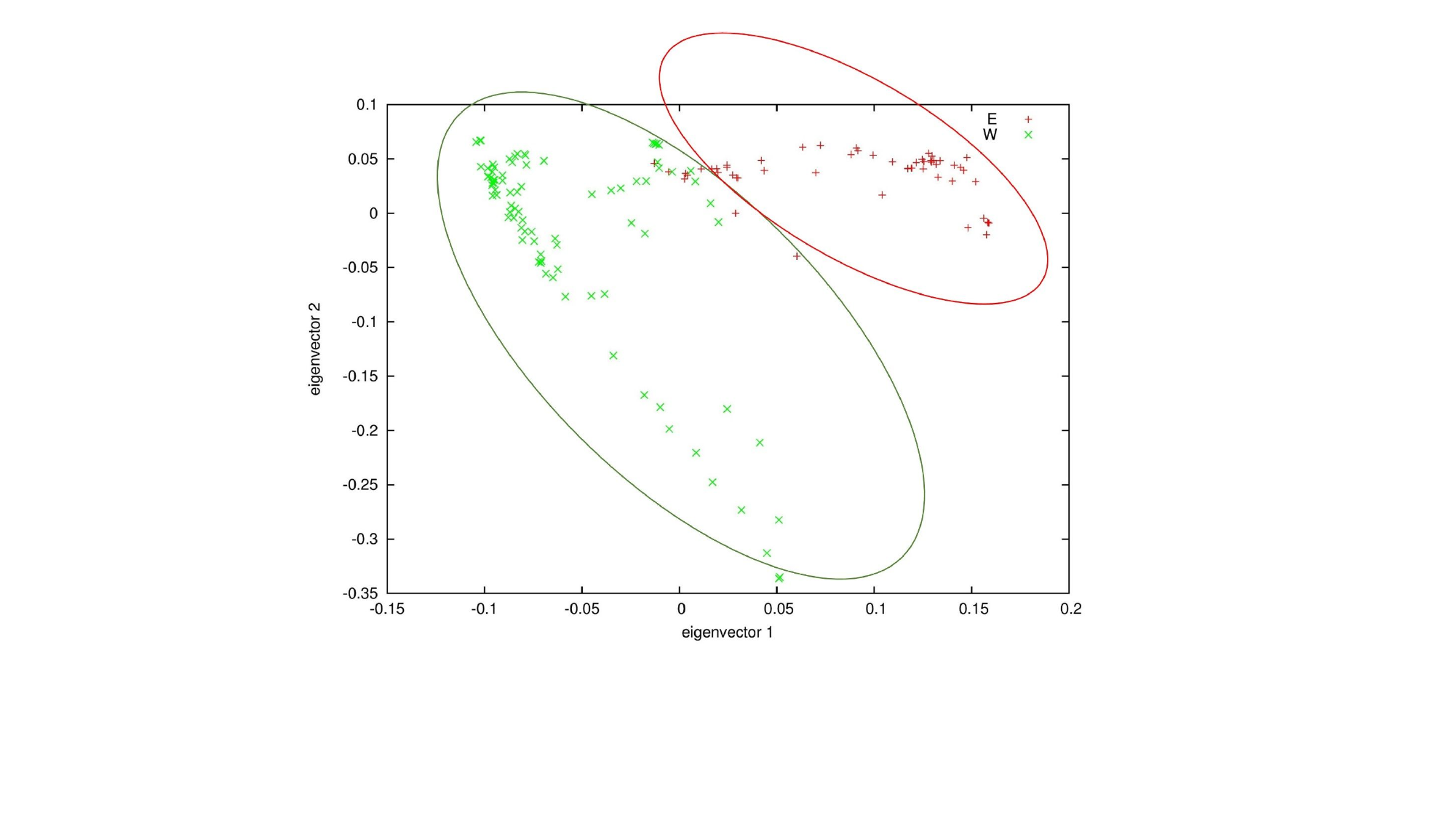

Supplement: Supplementary file 4 — Principal Component Analysis (PCA) for 142 accessions of wild narrow-leafed lupin colour-coded by phylogeny results (east/west Mediterranean) (PPTX 214 kb) [file 122_2017_3045_MOESM4_ESM.pptx]
