# Supplementary material for: Exploring the genetic and adaptive diversity of a pan-Mediterranean crop wild relative: narrow-leafed lupin
Source: Theor Appl Genet. 2018 Jan 20;131(4):887–901. doi: 10.1007/s00122-017-3045-7 (PMC5852200; doi:10.1007/s00122-017-3045-7)
Supplement: Supplementary file 5 — Supplementary material 5 (PPTX 220 kb) [file 122_2017_3045_MOESM5_ESM.pptx]

## Slide 1
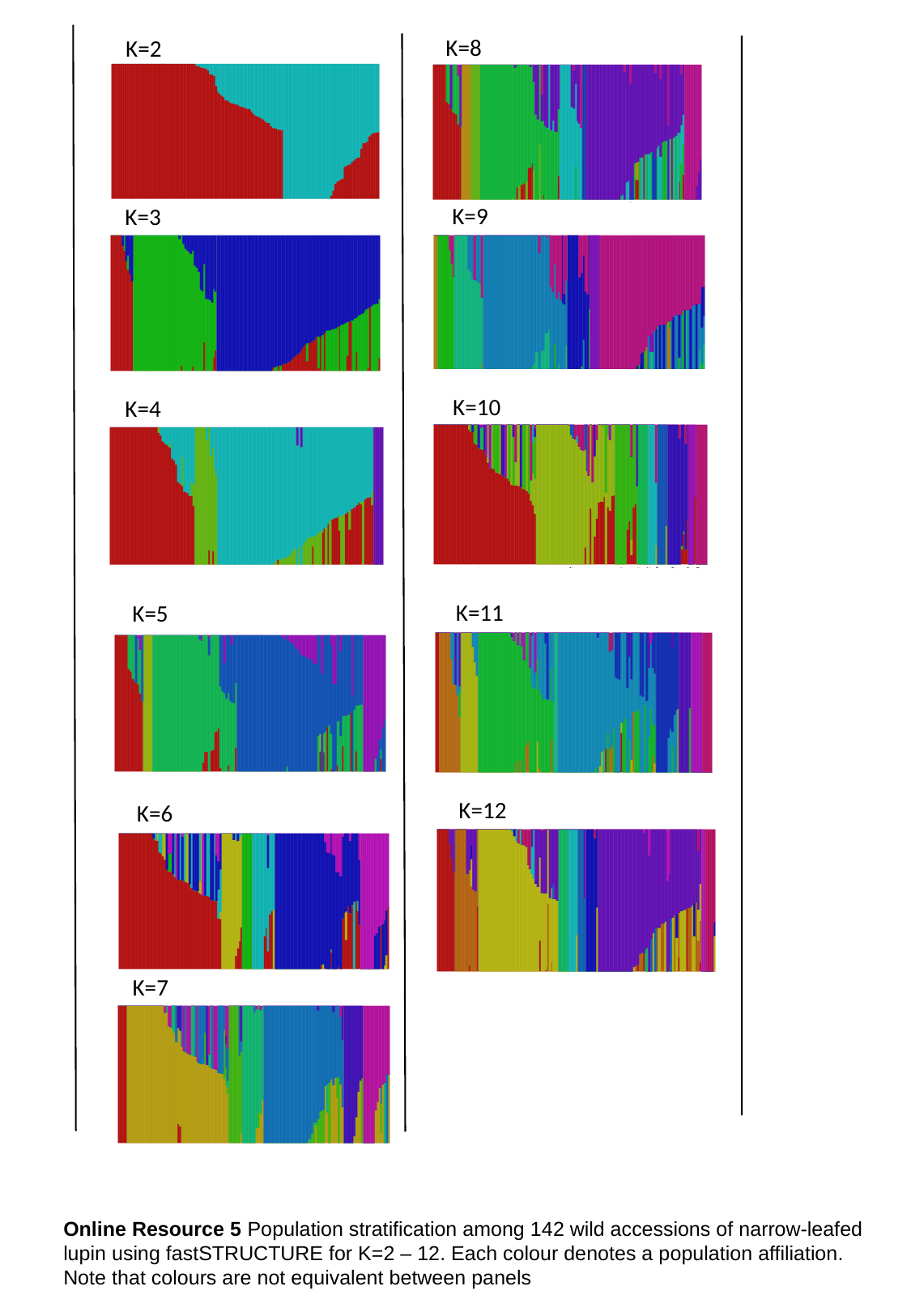

K=8
K=2
K=9
K=3
K=10
K=4
K=11
K=5
K=12
K=6
K=7
Online Resource 5 Population stratification among 142 wild accessions of narrow-leafed lupin using fastSTRUCTURE for K=2 – 12. Each colour denotes a population affiliation. Note that colours are not equivalent between panels
